# Staged or Simultaneous Surgery for Colon or Rectal Cancer with Synchronous Liver Metastases: Implications for Study Design and Clinical Endpoints

**DOI:** 10.3390/cancers15072177

**Published:** 2023-04-06

**Authors:** Sheraz Yaqub, Georgios Antonios Margonis, Kjetil Søreide

**Affiliations:** 1 Department of Hepatobiliary and Pancreatic Surgery, Oslo University Hospital, 0372 Oslo, Norway; 2 Institute of Clinical Medicine, University of Oslo, 0450 Oslo, Norway; 3 Department of Surgery, Memorial Sloan Kettering Cancer Center, New York, NY 10065, USA; 4 Department of Gastrointestinal Surgery, Stavanger University Hospital, 4011 Stavanger, Norway; 5 Department of Clinical Medicine, University of Bergen, 5021 Bergen, Norway

**Keywords:** colorectal cancer, liver metastases, simultaneous surgery, staged surgery, clinical endpoints

## Abstract

**Simple Summary:**

Patients who present with a primary colon or rectal cancer and are diagnosed with liver metastases at the same time (synchronous liver metastasis) have a number of treatment options when considering management. Providing systemic disease control alone involves several treatment considerations. For surgery, the decision rests with treating the primary tumor first, the liver metastasis first, or resection of both disease locations in one procedure. The decision rests on several conditions. Designing a trial to take all these factors into account while maintaining a reasonable endpoint for patients and caregivers is not straightforward. Here, we discuss some of the underlying points when considering simultaneous resection of colon or rectal cancer with liver metastases.

**Abstract:**

In patients presenting with colorectal cancer and synchronous liver metastases, the disease burden related to the liver metastasis is the driving cause of limited longevity and, eventually, risk of death. Surgical resection is the potentially curative treatment for colorectal cancer liver metastases. In the synchronous setting where both the liver metastases and the primary tumor are resectable with a relative low risk, the oncological surgeon and the patient may consider three potential treatment strategies. Firstly, a “staged” or a “simultaneous” surgical approach. Secondly, for a staged strategy, a ‘conventional approach’ will suggest removal of the primary tumor first (either colon or rectal cancer) and plan for liver surgery after recovery from the first operation. A “Liver first” strategy is prioritizing the liver resection before resection of the primary tumor. Planning a surgical trial investigating a two-organ oncological resection with highly variable extent and complexity of resection as well as the potential impact of perioperative chemo(radio)therapy makes it difficult to find the optimal primary endpoint. Here, we suggest running investigational trials with carefully chosen composite endpoints as well as embedded risk-stratification strategies to identify subgroups of patients who may benefit from simultaneous surgery.

## 1. Introduction

Colorectal cancer (CRC) is the third most common cancer worldwide and about one in five patients present with liver metastases at the time of diagnosis (synchronous liver metastases) [1,2]. In patients who present with synchronous disease, the disease burden related to the liver metastasis is the driving cause of limited longevity and, eventually, death. Surgical resection is the optimal potentially curative treatment for CRC liver metastases (CRLM) [3,4,5]. Although some patients present with symptoms from the primary tumor requiring urgent management (e.g., acute obstruction, perforation, or bleeding), the majority can be managed in an elective setting. Following an adequate staging with colonoscopy with biopsy and cross-sectional imaging, all patients should be discussed in a multidisciplinary tumor board meeting to plan the most appropriate treatment pathway. For patients with synchronous CRLM [6], there is substantial evidence that patients with a high tumor burden (multiple, large liver metastases requiring major liver surgery and/or locally advanced primary tumor) should start treatment with systemic chemotherapy, preferably based on a molecular analysis of the biopsy from the primary tumor, to inform the best oncological treatment according to cancer biology [7,8]. Surgery should be discussed on re-staging and re-evaluation after induction oncological therapy for these patients to arrive at the best treatment strategy.

**Figure 1:**
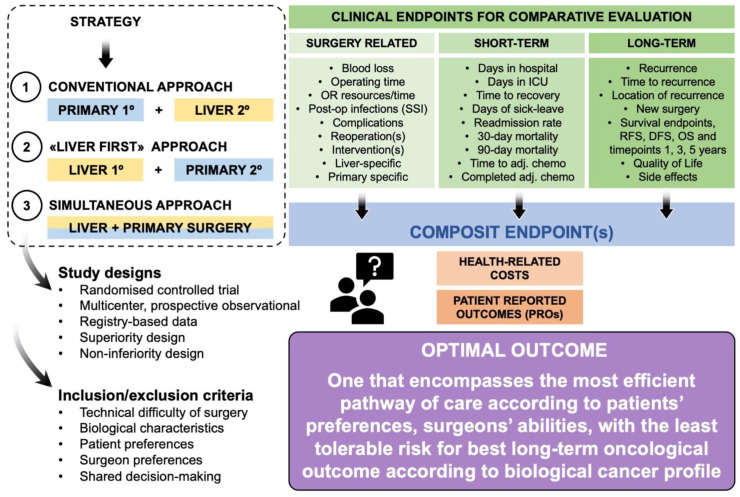
Schematic overview of different approaches for patients with colorectal cancer with synchronous liver metastases and clinical endpoints.

In the synchronous CRLM setting, where both the liver metastases and the primary tumor are resectable with a relative low risk within the standard surgical technical range and presumed tolerability by the patient fitness score and age (all of which are poorly defined criteria in the synchronous CRLM literature), the surgeon and patient can consider any three potential strategies (Figure 1). These are either a “staged” approach or a “simultaneous” approach. For staged strategies, a “conventional approach” will suggest removal of the primary tumor first (either colon or rectal cancer) and plan for liver surgery after recovery of the first operation. A “Liver first” or reversed strategy will prioritize the liver resection first [9,10]. Arguments have been put forward that a “liver-first” strategy is associated with improved survival [11], but such improved survival figures usually relate to those who actually are resected and “intention to treat” is often neglected in these comparative studies. Further, there is considerable heterogeneity to the use of perioperative chemotherapy in this setting, either as a strictly “neoadjuvant” approach in resectable disease or for downstaging/downsizing/conversion purposes to the liver disease. A simultaneous approach is reported to have similar outcomes in well-selected patients [12,13], but with a higher risk of post-operative mortality in complex simultaneous surgery.

When it comes to choosing the optimal surgical strategy for resection of the primary tumor and synchronous liver metastases, the jury is still out. The data available (Figure 1) are scarce to justify any one strategy over the other and are insufficient for use of open or minimally invasive surgery [12,14,15,16,17]. At present, there is only one prospective randomized trial (METASYNC) that has examined simultaneous and staged resection of CRC with synchronous liver metastases [18]. Over the course of 10 years, the study recruited around 100 patients across some 10 centers with no difference in perioperative complications between the groups reported. Unfortunately, this trial was underpowered and took a very long time to recruit. However, several case studies and patient cohorts support these findings—staged or simultaneous surgery in selected patients are reported to have similar outcomes, as demonstrated recently in the prospective observational CoSMIC study [19]. Although several surgeons have already adopted the approach of simultaneous surgery [20], it is still unclear if there is a clinical benefit of removing both the primary tumor and liver metastases in one singular surgical procedure. Several reasons for the lack of uniform agreement include the several different research designs used (Figure 1) with different inclusion and exclusion criteria of patients, a number of endpoints reported ranging from surgery-specific to short- and long-term survival, and health-related costs (Figure 1).

Beyond the obvious best interest and preference for a best achievable outcome to patients, the question is of particular interest for hepatobiliary surgeons, colorectal surgeons, and oncologists regularly attending tumor board meetings and deciding patient pathways. Simultaneous resection of the primary tumor and liver metastases has been described in numerous retrospective audits and meta-analyses [21]. The potential benefits of simultaneous resections are the eradication of the tumor burden in one procedure, a potential overall shorter procedure time (one versus two settings), a reduced hospital stay with the likely benefits on quality of life, and an expected reduction in the use of health care services compared to staged procedures. However, concerns about accumulating complications and oncological outcomes remain. The optimal selection criteria deciding for whom simultaneous resections are beneficial remain undetermined and large variations exist in clinical practice [22]. Based on the current evidence, simultaneous resection is best restricted to patients with a limited liver tumor burden [23,24].

Hence, in order to address the clinical equipoise between simultaneous vs. staged resection, we discuss here what would be the optimal outcome parameter in any given future trial and the potential endpoints of interest that could be considered in this setting of synchronous CRLM.

## 2. Current Knowledge Gaps

Based on a recent comprehensive review of the literature review [21], we identified several unanswered questions concerning simultaneous resection for synchronous CRLM. These included, with respect to simultaneous resection, a lack of data or confirmed definitions for:

- The number of liver metastases that is considered safe to resect;
- The number of liver resections or liver segments that is considered safe to resect;
- The size of the future liver remnant that is considered safe (for simultaneous surgery);
- The timing of staged resection (liver or primary tumor first);
- Whether chemotherapy should be administered before or after surgery (or if at all);
- Oncological endpoints (recurrence, time to recurrence, survival, etc.);
- Quality of life;
- Health economics.

**Figure 2:**
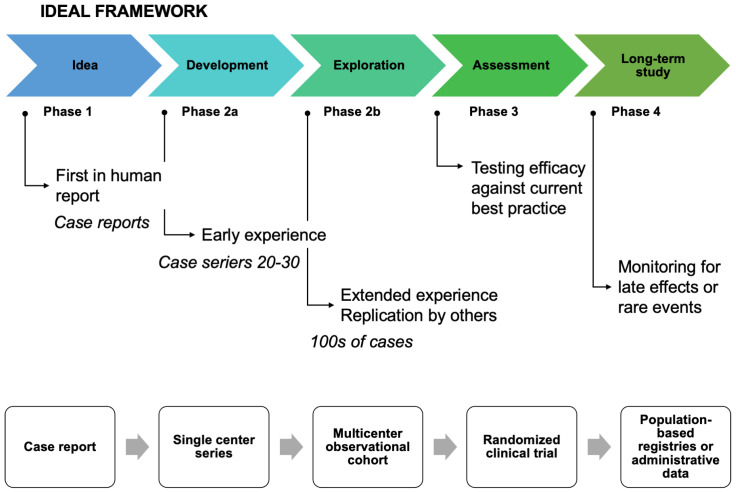
The IDEAL framework.

The lessons learned from current evidence is that some of these matters have to be addressed in well-planned multicenter randomized trials focusing on some of the above-mentioned questions and thereby filling the knowledge gaps in order to recommend simultaneous surgery to the appropriate patients. Based on Figure 2 and the IDEAL recommendations [25], there seem to be sufficient data for phase 1 and 2, scarce or lacking data for phase 3 which concerns randomized clinical trials on efficacy, and very little to an absence of phase 4 data from registries or population-based data.

## 3. Endpoints—Which One and for What Trial Design?

Planning a surgical trial investigating the complexity of a two-organ oncological resection with highly variable extent and complexity of resection as well as potential impact of perioperative chemo(radio)therapy makes it difficult to find the optimal primary endpoint. This has further impact on calculating the sample size and need of patient recruitment from a single center, nation-wide study, or international multicenter study, with each step increasing the complexity as well as reducing the number of variables you can demand from other centers. One should also realize the differences between pragmatic, real-life trials and trials that intend to answer a narrower research question with a more stringent superiority or non-inferiority design.

## 4. Surgery-Related Endpoints and Complications

The occurrence of intraoperative adverse events is related to post-operative complications and surgical outcome and, hence, may be an attractive endpoint [26]. Such endpoints could include intraoperative bleeding, accidental bowel perforation, rupture of splenic capsule during mobilization of left colon flexure, and injury to other organs resulting in conversion from minimally invasive surgery to laparotomy or resulting in the removal of a healthy organ (spleen, kidney, etc.). Length of surgery (time taken from “skin to skin”) may also be a variable of interest that may relate to the post-operative course.

However, several of these endpoints do not necessarily translate into clinical meaningful endpoints for the patients. While there may be an interest for the surgeons to measure a difference in operation time, blood loss, or accumulation of complications, these may only be of interest to the patient if it translates into a more severe or more cumbersome disease trajectory than otherwise expected. Hence, measuring a difference in the order of two-digit numbers of blood loss (e.g., 270 vs. 290 mL of estimated blood loss) between a staged and a simultaneous operation may show statistically significant results but represent no real clinical difference. While relatively easy to obtain, the surgery-related outcomes may not represent the endpoints that separate the pros and cons of these procedures. “All that can be measured, is not necessarily worth of measuring” as the saying goes.

## 5. Short-Term Outcomes

Short-term outcomes usually refer to the endpoints obtained during the initial stay (or up to 30 to 90 days after surgery) [27,28]. Endpoints of interest may include the accumulation of complications, resources needed (e.g., interventions, reoperation, transfer to high-dependency unit, or intensive care), and the recovery of the patient (e.g., time to mobilization, time to first bowel movement, or time spent in hospital, etc.).

Organ-specific complications are of particular interest and should be covered. Some examples include anastomotic leaks for colorectal surgery, presence of post-hepatectomy liver failure, or biliary leak after liver surgery.

In more general terms, complications can also be measured by the Clavien–Dindo score, Accordion, or the Comprehensive Complication Index (CCI). As in-hospital and even 30-day mortality is increasingly rare in modern colorectal and liver surgery, these endpoints are difficult to justify for comparison. Hence, composite endpoints are suggested to allow for comparison in liver surgery [29,30] and may be especially considered if a trial is planned. These are surrogate endpoints for the «real» outcome (post-operative survival) but may be used to make any given trial a feasible and obtainable time perspective. Power-sampling for post-operative mortality in this particular setting (synchronous CRLM) would make a required number of trial participants prohibitive and unattainable.

## 6. Long-Term Outcomes and Survival

Long-term endpoints could be patient-oriented, societal-oriented, and survival. A patient-oriented endpoint could be if the patient completes planned adjuvant oncological treatment or planned stoma reversal. A potential societal-oriented outcome could be cost effectiveness [31,32]. Sustainable use of resources is one of the UN Sustainable Development Goals. Therefore, any trials or study analyses that balance costs and health outcomes are warranted. Cost-effectiveness analyses are typically applied for reimbursement decisions for pharmaceuticals but are used less for medical devices and surgical procedures. Evaluation of altered clinical pathways could be a potential outcome. The hard endpoints regarding both disease-free survival (DFS) and overall survival (OS) take a long time (from 3 to 5 years and can be up to 10 years). Among those, OS might be preferable as a recent study suggested that DFS might be a suboptimal endpoint compared to OS in the setting of CRCLM [33]. Furthermore, around 70% of patients resected for CRCLM developed recurrence. Treatment of recurrence with surgery, chemotherapy, targeted therapy, or a combination of these modalities will surely affect OS.

## 7. Why a Composite Endpoint?

As discussed above, a single given endpoint, be it surgery-related or a short-term endpoint, may not sufficiently capture the patient pathway nor be frequent enough to power a study for the outcome with a reasonable or obtainable sample size. Hence, for procedures with rare outcomes or complications, a composite endpoint may be considered. This has been demonstrated for liver surgery in the past and has more recently been defined as so-called “textbook outcomes” [29,30].

## 8. Other Related Endpoints to Consider

A trial studying simultaneous surgery for synchronous CRLM should include endpoints focusing on the user (patients). There are several questionnaires developed to assess the quality of life (QoL) of cancer patients. The European Organisation for Research and Treatment of Cancer (EORTC) provides several validated QoL questionnaires which are disease-specific and may be considered as an element of the study in any given interventional trial on cancer patients. Furthermore, the EuroQol group has developed instruments to assess different dimensions of disease stage. Specifically, the EQ-5D-5L instrument comprises five dimensions: mobility, self-care, usual activities, pain/discomfort, and anxiety/depression. EQ-5D index values are used in the estimation of quality-adjusted life year (QALY) gains in economic evaluations of healthcare interventions. EQ-5D-5L also provide additional evidence on relative effectiveness of healthcare interventions. The following tools could be considered as secondary endpoints:

- EORTC-QLQ-30 (designed to measure cancer patients physical, psychological, and social functions);
- QLQ-CR29 (QoL measurement disease-specific for colorectal cancer);
- QLQ-LM21 (QoL measurement disease-specific for CRLM);
- EQ-5D-5L (measuring health-related QoL).

Another endpoint to be considered is the patients’ preference [34].

## 9. Who Should Be Included in a Surgical Trial?

The number of treatment alternatives for synchronous CRLM is staggering even if only considering the number of options for surgery of the primary (colonic versus rectal surgery) in combination with liver surgery (ranging from a simple wedge to major resections, with or without augmenting the future liver remnant; alone, or in combination with ablation procedures), using open or minimally invasive techniques with or without neoadjuvant/perioperative chemotherapy options [13,35]. On top of this comes selection of the appropriate candidate. Hence, comparability across studies is poor as no standard or uniform inclusion criteria have been used. For one, molecular profiling of the cancer biology has only been performed in more recent years, thus, older studies do not include this information. Current knowledge refers tumor biology to oncological outcomes, with good, bad, and ugly tumors [36].

A point to be noted in this matter is that the indication for surgery of CRCLM varies greatly between institutions. Some surgeons and institutions may be more aggressive in recommending liver surgery for metastases while others may be more conservative. This may affect the results of multicenter trials as well as the rate of patient recruitment in a trial.

### 9.1. Staged or Simultaneous Surgery?

Simultaneous surgery for rectal cancer is controversial [37,38,39,40]. Some argue that it can be performed safely, even in combination with major liver surgery [40]. However, as rectal cancers have a more complex treatment algorithm, with a higher risk for complications, many have deferred simultaneous surgery for patients with rectal cancer. In particular, for those with locally advanced disease requiring neoadjuvant chemoradiation for down-staging, the risk has been viewed too high by some. Furthermore, there have been reports arguing safety of liver-first strategy in locally advanced rectal cancer with synchronous liver metastases [10]. However, some reports even suggest liver surgery simultaneous with extensive pelvic surgery [37,38].

### 9.2. Tumor Biology and Molecular Markers—Any Role?

Two studies have included KRAS mutational analyses in the outcome. In the CoSMIC study, no influence of KRAS status was found in the 83 patients investigated [41]. However, in a large, multicenter study from China, there was a benefit from simultaneous resection of synchronous CRLM restricted to patients with a KRAS wild-type tumor [16]. The findings need to be corroborated in other studies but suggest that cancer biology, as marked by the molecular predominant features, plays an important part in the long-term outcomes. This may be a step toward precision surgery.

## 10. Conclusions

As universally accepted stratification inclusion criteria are lacking and with a mix of endpoints used in the existing literature, the optimal role of simultaneous surgery of primary colorectal cancer and synchronous liver metastases is not known. There is a selection in the literature towards minor liver resections and non-symptomatic primary colon cancers derived from experience of higher complication rates for more complex combinations of procedures. Hence, data may not necessarily be extrapolated from one cohort to the other. We would suggest running investigational trials with carefully chosen composite endpoints as well as embedded risk-stratification strategies to identify subgroups of patients who may benefit from simultaneous surgery.

## Data Availability

Not applicable.
